# ﻿Circumscription of the *sclerolobii* group of *Amblycerus* Thunberg (Coleoptera, Chrysomelidae, Bruchinae) with descriptions of four new species and a revision of the species groups of the genus

**DOI:** 10.3897/zookeys.1252.144951

**Published:** 2025-09-19

**Authors:** Cibele Stramare Ribeiro-Costa, Geoffrey E. Morse

**Affiliations:** 1 Universidade Federal do Paraná, Caixa Postal 19020, 81531-980, Curitiba, Paraná, Brazil Universidade Federal do Paraná Curitiba Brazil; 2 Department of Biology, University of San Diego, 5998 Alcalá Park, San Diego, CA 92110, USA University of San Diego San Diego United States of America

**Keywords:** Amblycerina, Neotropical region, seed beetles, systematics, Western Hemisphere

## Abstract

The seed-beetle *Amblycerus* Thunberg, 1815 the second most diverse genus of Bruchinae (Chrysomelidae) in the Western Hemisphere, is organized into groups of species, as many others in the subfamily. The main objective of this work is to delimit the group *sclerolobii*, here established as composed by five described species, *A. sclerolobii* Ribeiro-Costa, 2000, *A. kingsolveri* Ribeiro-Costa, 1993, *A. marinonii* Ribeiro-Costa, 1993, *A. manauara* Ribeiro-Costa, 2000, *A. tachigaliae* Kingsolver, 1976 and four new species: *A. biacutus* Ribeiro-Costa, **sp. nov.**, *A. falcorostrus* Ribeiro-Costa & Morse, **sp. nov**., *A. morsei* Ribeiro-Costa, **sp. nov.** (type localities: Brazil: Amazonas, Manaus) and *A. truncatus* Ribeiro-Costa, **sp. nov.** (type locality Brazil: Mato Grosso, Sinop). Therefore, including these new species 70 Brazilian species of *Amblycerus* were recorded. *Amblycerus bicolor* (Pic, 1927) previously considered to belong to this group is excluded and placed as incertae sedis. The *sclerolobii* group is distributed mainly in the Brazilian Amazonia biome, consuming *Tachigali* Aubl., *Dinizia* Ducke, and *Stryphnodendron* Mart. seeds (Fabaceae). Diagnoses are presented for this group and for ten species (including *A. bicolor*), and descriptions for the four new ones, an identification key for the nine species, colored photographs of external morphology, and detailed male genitalia illustrations for all the species studied. Due mainly to previously broad contributions on the taxonomy of *Amblycerus* that resulted in many species groups, we also present a revised study of this intrageneric taxonomic level. From 37 species groups now we update to 32 groups with a total of 96 species assigned to them. Finally, a detailed systematic study of some specific groups is also suggested before mergence.

## ﻿Introduction

Seed beetles (Chrysomelidae: Bruchinae) currently encompasses approximately 1,750 described species worldwide (except Antarctica) and are assigned into 65 genera (Morse and Ribeiro-Costa 2024). Two genera stand out in the Western Hemisphere, *Amblycerus* Thunberg, 1815 and *Acanthoscelides* Schilsky, 1905. Between 1990 and now, 32 *Amblycerus* species were described by the first author and her collaborators and mainly recorded from Brazil (Ribeiro-Costa et al. 2018), although the number of new Brazilian species seems inexhaustible and there are many to be discovered in other countries. Even with the description of a significant number of new taxa, *Amblycerus* is still the second largest genus of Bruchinae in the Western Hemisphere, after *Acanthoscelides*. While *Amblycerus* still needs a broad phylogenetic study to confirm its monophyly, it is unified by multiple characters (Kingsolver 2004); *Acanthoscelides*, however, is likely paraphyletic and lacks precise delimitation (Kergoat and Silvain 2004; Kergoat et al. 2005, 2008). Regardless of the diversity of both of these genera, previous authors have made efforts to organize each into definable species groups (Johnson 1983, 1989, 1990; Ribeiro-Costa 1995; Romero-Nápoles et al. 1996). This procedure was applied in multiple revisionary works of Bruchinae from the Western Hemisphere (e.g., *Caryedes* Hummel, 1827 by Kingsolver and Whitehead 1974, *Ctenocolum* Kingsolver & Whitehead, 1974 by Albuquerque et al. (2014), *Gibbobruchus* Pic, 1913 by Manfio et al. 2013, and *Sennius* Bridwell, 1946 by Johnson and Kingsolver 1973).

Focusing on Brazilian *Amblycerus*, Ribeiro-Costa (1995) organized 56 species in 22 groups based on a phenetic analysis. One year later, Romero-Nápoles et al. (1996) treated 40 species distributed in the United States and Mexico and placed them into 15 groups that they intuitively proposed. Romero-Nápoles et al. (2002) analyzed the same species revised in their previous paper based on a cladistic analysis of morphological characters and found that, of the 15 previously defined groups in their revisionary study (Romero-Nápoles et al. 1996), only nine clades corresponded to similar taxa.

Considering that these studies were developed almost concurrently (Ribeiro-Costa 1995; Romero-Nápoles et al. 1996) and that groups were not discussed between authors, comparisons of their composition is undoubtedly appropriate. In addition, over the years new species were added into groups (Ribeiro-Costa et al. 2014), being essentially an updated list of the current groups for future studies in *Amblycerus*.

It is clear that the groups/clades of *Amblycerus* are difficult to define, as numerous morphological features appear to have evolved independently in different lineages; therefore, there is a need for a cladistic morphological and/or molecular analysis with representatives from throughout the Western Hemisphere. We are aware many species treated by Romero-Nápoles et al. (1996) from the United States and Mexico as well as some of the material treated in Ribeiro-Costa (1995) from Brazil have a wide distribution extending also in Central America. It is worth noting that Ribeiro-Costa (1995) included some Central American species in her Brazilian groups that were not included in her analysis due to their close morphological similarities with one or another Brazilian species. But, until now, no broad revisionary taxonomic study was developed on *Amblycerus* focusing on the fauna from the Central and other South American countries. Consequently, there is a gap to be filled and this knowledge may elucidate the current formation of groups, the affinity among them, and probably new species to be discovered.

Even with these difficulties and considering the high diversity of the genus, it is essential that we continue describing species and trying to organize species into groups as previous authors have done. Our aims are to provide valid names, identification keys, colored images that have not been presented in literature before (Ribeiro-Costa 1995; Romero-Nápoles et al. 1996, 2002), and detailed male genitalia illustrations for precise species identifications. All of these procedures will also reduce the gaps in understanding the diversification of the genus *Amblycerus* when a phylogeny will be available for the entire genus.

During recent studies of the Brazilian *Amblycerus* fauna we recognized four undescribed *Amblycerus* species, one of them provided by the second author. These species were identified as belonging to the *sclerolobii* group, one of the 22 groups suggested by Ribeiro-Costa (1995). This group is composed of five previously described species: *Amblycerus sclerolobii* Ribeiro-Costa, 2000, *A. kingsolveri* Ribeiro-Costa, 1993, *A. marinonii* Ribeiro-Costa, 1993, *A. manauara* Ribeiro-Costa, 2000, and *A. bicolor* (Pic, 1927), with *A. bicolor* the least joined to the others in the phenetic diagrams. As these species are not distributed in the United States and Mexico, and they were not included in Romero-Nápoles et al. (1996, 2002). The hosts of all of the species with known host associations belong to rainforest trees in the Caesalpinoideae clade of the Fabaceae (Bruneau et al. 2024).

This work has the aim to carry out a detailed morphological study of the *sclerolobii* species group, and identify any other species that may be included from other countries not evaluated by the previous authors (Ribeiro-Costa 1995; Romero-Nápoles et al. 1996), and to then provide a formal delimitation of the *sclerolobii* group that is lacking to date. We then take the opportunity to revise all *Amblycerus* groups and list the species composition of each one with their rearrangements and possible congruences.

## ﻿Materials and methods

The material examined was loaned from the museums/collections listed below, except the specimens of *A. tachigaliae* Kingsolver, 1976 from Brazil for which we received only labels data from TAMU. Acronyms of museums/collections and curators’ names are also provided and correspond to those specified in Evenhuis (2024).

**DZUP**Coleção de Entomologia Pe. J. S. Moure, Departamento de Zoologia, Universidade Federal do Paraná, Curitiba, Paraná, Brazil (C. S. Ribeiro-Costa);

**INPA**Coleção de Invertebrados, Instituto Nacional de Pesquisas da Amazônia, Manaus, Amazonas, Brazil (A. L. Henriques);

**MNRJ**Museu Nacional, Universidade Federal do Rio de Janeiro, Rio de Janeiro, Brazil (M. A. Monné);

**MZSP**Museu de Zoologia da Universidade de São Paulo, São Paulo, Brazil (S. Casari);

**SDMC** University of San Diego, San Diego, California, United States of America (research collection of G.E. Morse with ultimate deposition in the San Diego Natural History Museum, San Diego, California, United States of America);

**TAMU**Texas A&M University Insect Collection, College Station, Texas, United States of America (J. Oswald and M. Cupello);

**USNM**United States National Museum of Natural History, Washington D.C., United States of America (A. Konstantinov and E. Roberts).

For the data of material examined, the labels were copied verbatim and a semicolon (;) separates different labels, backslashes (\) separates different lines in the same label, and text within square brackets are extra comments.

Most characters were observed from dry and pinned insects. The methods for extracting and clearing male genitalia followed Manfio et al. (2013). The illustrations of *Amblycerus sclerolobii*, *A. manauara*, *A. kingsolveri*, and *A. marinonii* were done as detailed in Ribeiro-Costa (1997) and are here modified from Ribeiro-Costa (1993, 2000). The illustrations of *Amblycerus truncatus* Ribeiro-Costa, sp. nov., *A. biacutus* Ribeiro-Costa, sp. nov., *A. falcorostrus* Ribeiro-Costa & Morse, sp. nov., *A. morsei* Ribeiro-Costa, sp. nov., *A. tachigaliae*, and *A. bicolor* followed the same methods of Ribeiro-Costa (1997), in which with the contours of the median lobe, the internal sac and each sclerite were obtained in general separately, and then a composition was made by plotting sclerites in layers from bottom to top in the internal sac. The equipment used for these procedures were Leica MZ6 stereomicroscope then Olympus BX50 microscope of the DZUP.

Colored images of the external morphology were captured in two ways. In the laboratory of the first author images of the external morphology were obtained using a Nikon D610 digital camera attached with a Nikon Macro Photo Lens AF-S Micro Nikkor 40mm f/2.8 coupled to Carl Zeiss Stemi 2000-C. The number of images taken per specimen were taken according to the depth of the specimen/structure, from lower to the upside. Images with no parts focused were excluded previously to proceed merging using the software Combine ZM 1.2 (Alan Hadley, United Kingdom). In the laboratory of the second author, they were captured using stacking technology developed by Macroscopic Solutions (Tolland, CT) using a Canon EOS 6D digital camera attached to a Canon Extension Tube EF25 II paired with a Canon Macro Photo Lens MP-E 65-mm 1×–5×. The number of images taken per specimen were optimized according to the depth of the specimen, and the resulting photographs were compiled using ZereneStacker v. 1.04 (Zerene Systems LLC, Richland, WA, USA) and saved and edited in JPEG format using the Adobe Photoshop 2023 application suite (Adobe Inc., San Jose, CA, USA).

The terminology adopted was that of Ribeiro-Costa et al. (2014), including terms for some thoracic and abdominal sclerites from Lawrence et al. (2010).

Host plant names are updated according to the Plants of the World Online Database from Kew Gardens (Powo 2024). The specific name *A. sclerolobii* was proposed in reference to the holotype host plant, *Sclerolobium* sp. Here we use the same name, *sclerolobii*, to formally establish the group *sclerolobii* to maintain the nomenclature stability. However, it is worth noting that the valid genus name of the host plant is *Tachigali* (Huamantupa-Chuquimaco et al. 2024). Host plant associations of all *Amblycerus* species groups will be explored in future studies.

Measurements were done using an ocular micrometer attached to a Carl Zeiss Discovery V8. We measured for dimensions up to ten specimens when available, although for indices one specimen of each species was randomly chosen following traits as demarcated in Ribeiro-Costa (1997:631; Figs 1, 2, 5, 8). Abbreviations were: **BL**, body length (from anterior margin of pronotum to elytra apex); **BW**, body width (the largest width). For the internal sac of male genitalia, the abbreviations were: **BR**, basal region; **MR**, median region; **AR**, apical region. Localities with asterisk (*) indicate new records.

## ﻿Results and discussion

The text was divided in two sections: Taxonomy and Revision of Amblycerus species groups. In the section Taxonomy we present the descriptions of the new species as they are being keyed, and subsequently the diagnosis of the described species also in the same order they appear in the key. The same species sequence was followed in Table 1 and the plates that were organized by each species individually to facilitate precise species identification.

**Table 1.** Species of the *sclerolobii* group of *Amblycerus* studied in this paper and the excluded species, their distribution and host plants. (-) Excluded species, (*) New record.

Species	Distribution	Host plants
*A. truncatus* sp. nov.	Brazil (Mato Grosso-Sinop)	*Dimorphandra macrostachya* (Fabaceae)
*A. falcorostrus* sp. nov.	Brazil (Amazonas-Manaus)	Unknown
*A. biacutus* sp. nov.	Brazil (Amazonas-Manaus)	*Stryphnodendron* sp. (Fabaceae)
*A. morsei* sp. nov.	Brazil (Amazonas-Manaus)	*Dinizia excelsa* (Fabaceae)
*A. manauara*	Brazil (Amazonas-Manaus)	Unknown
*A. sclerolobii*	Brazil (Amazonas-Estirão do Equador, Minas Gerais-Viçosa)	*Tachigali denudata* (Fabaceae), *Tachigali* sp.
*A. tachigaliae*	Panama (Barro Colorado Isl., Colón*), Brazil* (Amazonas- Manaus)	*Tachigali versicolor* (Fabaceae)
*A. kingsolveri*	Brazil (Amazonas-Manaus-Rio Tarumã Mirim)	Unknown
*A. marinonii*	Brazil (Mato Grosso-Cuiabá, Goiás-Dianópolis, Barro Alto*)	*Tachigali rubiginosa* (Fabaceae)
*A. bicolor*	Brazil (Para), Suriname, French Guiana	Cassia sp. (Fabaceae)

### ﻿Taxonomy

The *sclerolobii* group, first suggested by Ribeiro-Costa (1995), is here modified by the exclusion of *Amblycerus bicolor* (Fig. 11), the phenetically distant species of the others of this group. This species shares characters of the group *sclerolobii*, including frons convex, the elytral vestiture composed of unicolorous setae, and elytra depressed, flat at middle (Ribeiro-Costa 1995). However, Ribeiro-Costa (1995) pointed out that the internal sac sclerites, the scutellum (Fig. 11C), and the color pattern of integument on the pygidium could exclude this species from the *sclerolobii* group. Here we agree with these comments and add more differences: pronotum with elongate coarse punctures on lateral areas (Fig. 11B); prosternal process very narrow, laminar; apex of elytra and pygidium with coarse punctures. In addition, considering the internal differences of male genitalia, we highlight two unpaired sclerite at MR (Fig. 11G), one anterior with denticles and the posterior long, entire, not as a wishbone-shaped sclerite similar to the species of the *sclerolobii* group which has separate stems. Until further character analyses and more complete sampling of species in the genus are developed, *A. bicolor* is considered here excluded from the *sclerolobii* group and placed incertae sedis.

Analyzing material from Panamá, we observed that the species *A. tachigaliae*, not included in the previous works of Ribeiro-Costa (1995) and Romero-Nápoles et al. (1996) because of its distribution, shares many external characters with the *sclerolobii* group that justify, at present, its inclusion in this group. We note mainly the general integument color, body vestiture homogeneous and unicolorous; pygidium with faint dense median line of setae; elytra flat medially; pronotum with moderate coarse punctures only on lateral areas; scutellum tridentate, all teeth the same size (Fig. 10). The pattern of sclerites of the internal sac of male genitalia do not show the same affinity with the pattern shown by the *sclerolobii* group; there is an extra pair of MR sclerite (Fig. 10G) in addition to the laminar sclerites, common to the *sclerolobii* group. This kind of discordance, pattern of vestiture versus pattern of genitalia, was also observed in *A. manauara* (Fig. 9), whose internal characters of male genitalia showed differences, mainly in the form of dorsal and ventral valve (Fig. 9F), compared to the others of the group *sclerolobii*. Therefore, to a lesser extent, but in the same way of *A. bicolor*, only after a broader analysis will it be possible to better understand the positioning of these species.

#### ﻿Group *sclerolobii*

Figs 1–10; Table 1

**Species composition.** This group is composed of the five described species: *A. sclerolobii*, *A. kingsolveri*, *A. marinonii*, *A. manauara*, *A. tachigaliae*, and four new species: *A. biacutus* Ribeiro-Costa, sp. nov., *A. falcorostrus* Ribeiro-Costa & Morse, sp. nov., *A. morsei* Ribeiro-Costa, sp. nov. and *A. truncatus* Ribeiro-Costa, sp. nov. The *sclerolobii* group is distinguished from other *Amblycerus* species groups by combinations of characters listed in the diagnosis.

**Figure 1.**
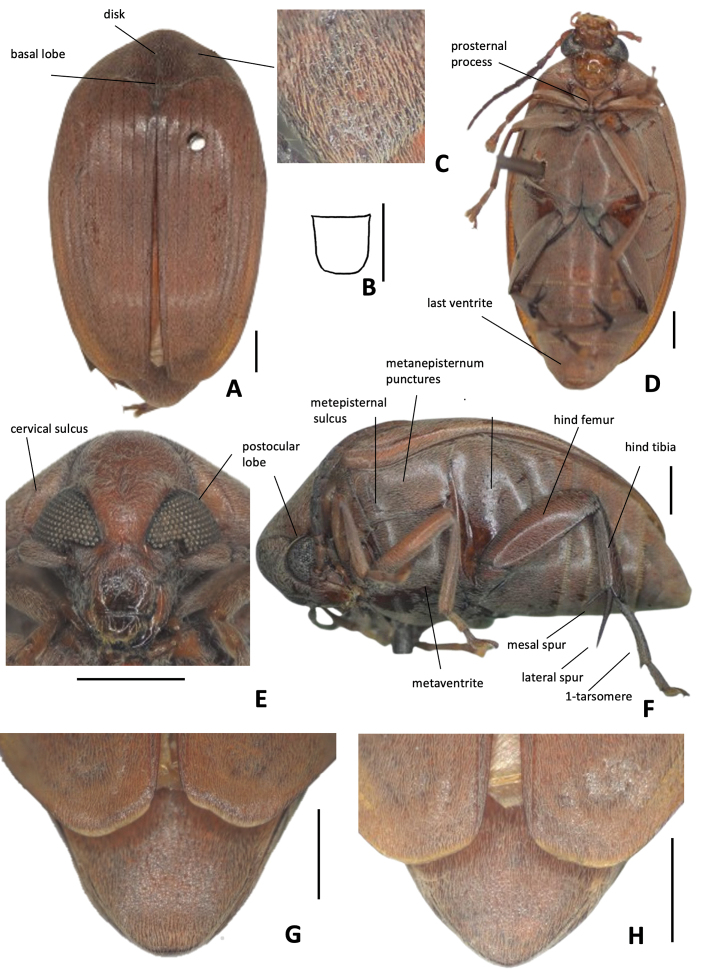
*Amblycerus truncatus* Ribeiro-Costa, sp. nov.: **A** dorsal view **B** scutellum **C** punctures on lateral areas of pronotum **D** ventral view **E** head frontal view **F** lateral view **G** male pygidium **H** female pygidium. Scale bars: 1.0 mm.

**Diagnosis.** Integument homogeneous, without black spots, usually reddish brown on dorsum contrasting with dark brown to black on thoracic ventrites and legs. Dorsum covered with yellowish pubescence evenly distributed, without stripes or variegated pattern, sometimes near elytral sutures with whitish setae; pygidium usually with a faint dense median line of setae. Head without frontal carina, frons convex. Eye coarsely faceted, strongly prominent laterally, postocular lobe very narrow. Disc of pronotum with moderately coarse punctures only on lateral areas (Fig. 1C); prosternal process narrow, gently expanded beyond anterior coxae (Fig. 1D). Scutellum usually tridentate with all teeth the same size (Figs 3B, 4B, 5B, 6B, 7C, 8B, 9B, 10B), rarely truncate (Fig. 1B). Elytra depressed, flat medially, without coarse punctures on interstriae. Metanepisternum and metaventrite without coarse punctures (Fig. 1F), metepisternal sulcus usually as a right angle (Fig. 1F). Hind coxae with moderately coarse, sparse punctures (Fig. 1F). Pygidium with moderately coarse and dense punctures. Male genitalia, median lobe: dorsal valve usually subtriangular, lateral margins almost straight or gently curved, apex usually rounded. Internal sac, AR with a unique pair or two pairs of sclerites, MR with a pair of laminar sclerites usually with serrate margin and an unpaired wishbone-shaped sclerite.

**Comparative notes.** The *sclerolobii* group differs from the other groups proposed to date by several characters treated together (see Diagnosis). Analyzing the diagnostic characters of the group with the characters from the partial phylogeny of *Amblycerus* (Romero-Nápoles et al 2002) we could recognize the *sclerolobii* group shares the synapomorphy of two long, serrate blades (or laminar sclerites) in the internal sac of the male genitalia with the subclade *A. barcenae* + *A. pictus* that is part of the *marmoratus* clade with four other species. However, it differs by not having a strongly mottled pattern of pubescence on the dorsum, which is indicated by Romero-Nápoles et al. (2002) as a synapomorphy of the group.

It is interesting to draw attention that these two long serrate, laminar/blade sclerites occur in several species within the genus (Ribeiro-Costa 1995); however, they vary in general shape, length of the serrated margin, even whether they are straight or winding. Maybe the *sclerolobii* group should be included in a broader clade, which encourages further scientific investigation of this *Amblycerus* group.

Contrary to *A. barcenae* and *A. pictus*, the *pterocarpae* group, herein reorganized and comprising *A. pterocarpae* and *A. luciae*, externally resembles the *sclerolobii* group because of the homogeneous pattern of vestiture on the dorsum. However, the pattern of male sclerites in the internal sac easily separates these both groups.

**Figure 2.**
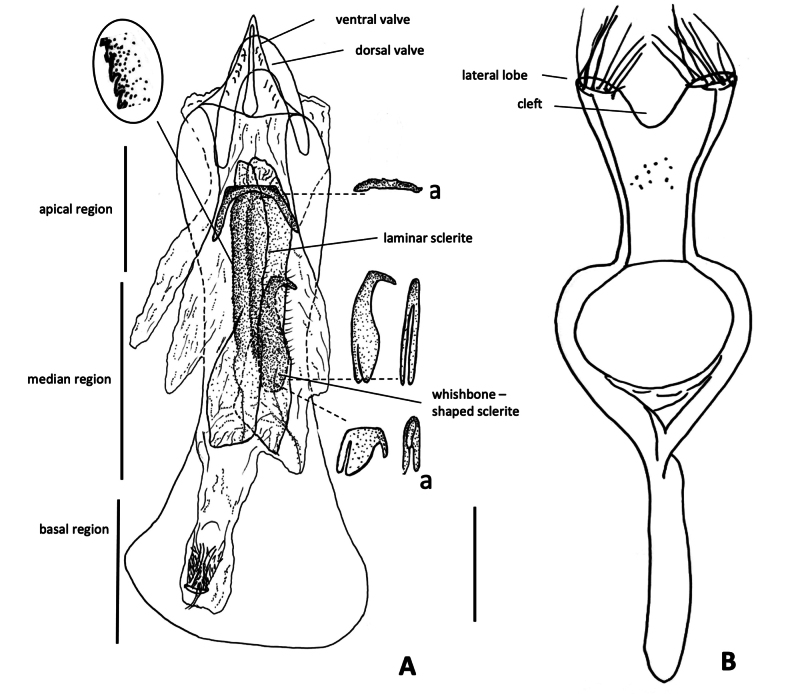
*Amblycerus truncatus* Ribeiro-Costa, sp. nov.: **A** median lobe of male genitalia **B** tegmen of male genitalia **a** intraspecific variation. Scale bar: 0.5 mm.

**Figure 3.**
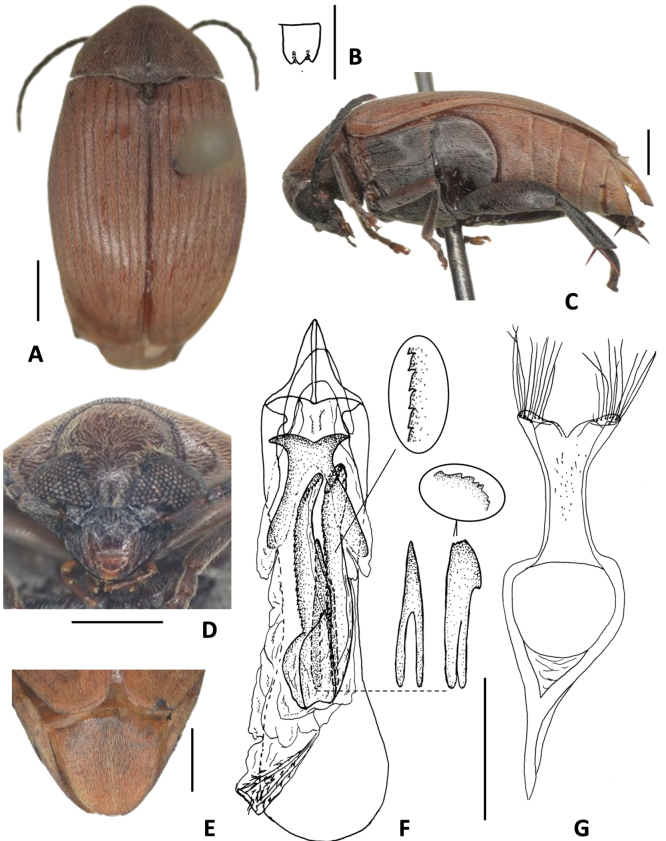
*Amblycerus falcorostrus* Ribeiro-Costa & Morse, sp. nov.: **A** dorsal view **B** scutellum **C** lateral view **D** head frontal view **E** male pygidium **F** median lobe of male genitalia **G** tegmen of male genitalia. Scale bars: 1.0 mm (**A, C–E**); 0.5 mm (**B, F, G**).

**Figure 4.**
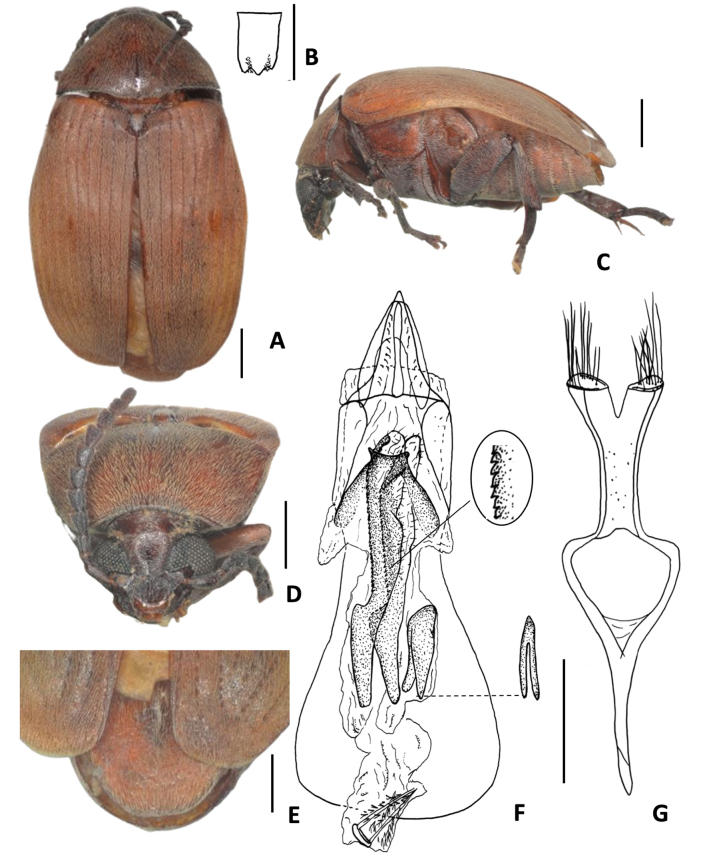
*Amblycerus biacutus* Ribeiro-Costa, sp. nov.: **A** dorsal view **B** scutellum **C** lateral view **D** head frontal view **E** male pygidium **F** median lobe of male genitalia **G** tegmen of male genitalia. Scale bars: 1.0 mm (**A, C–E**); 0.5 mm (**B, F, G**).

**Figure 5.**
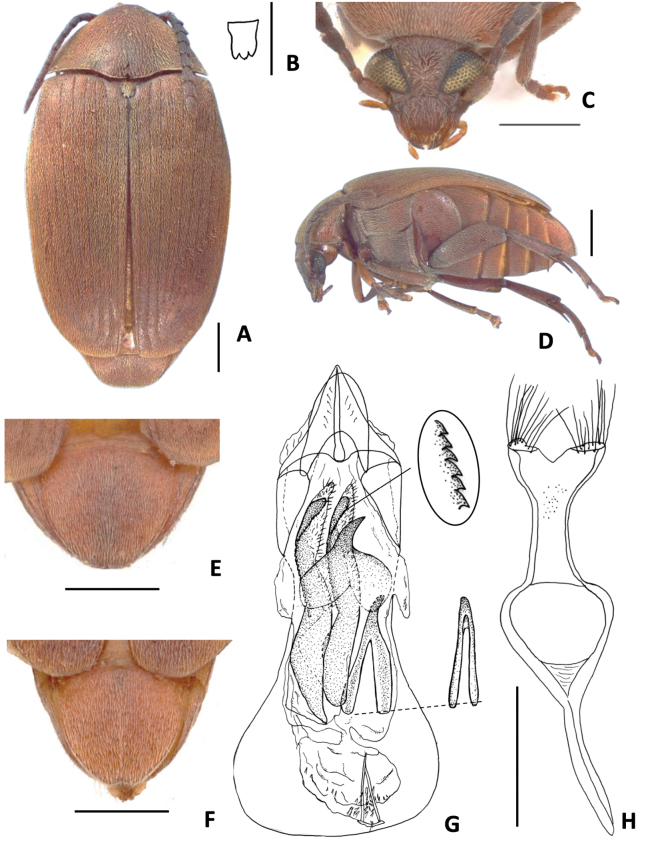
*Amblycerus morsei* Ribeiro-Costa, sp. nov.: **A** dorsal view **B** scutellum **C** head frontal view **D** lateral view **E** male pygidium **F** female pygidium **G** median lobe of male genitalia **H** tegmen of male genitalia. Scale bars: 1.0 mm (**A, C–F**); 0.5 mm (**B, G, H**).

**Figure 6.**
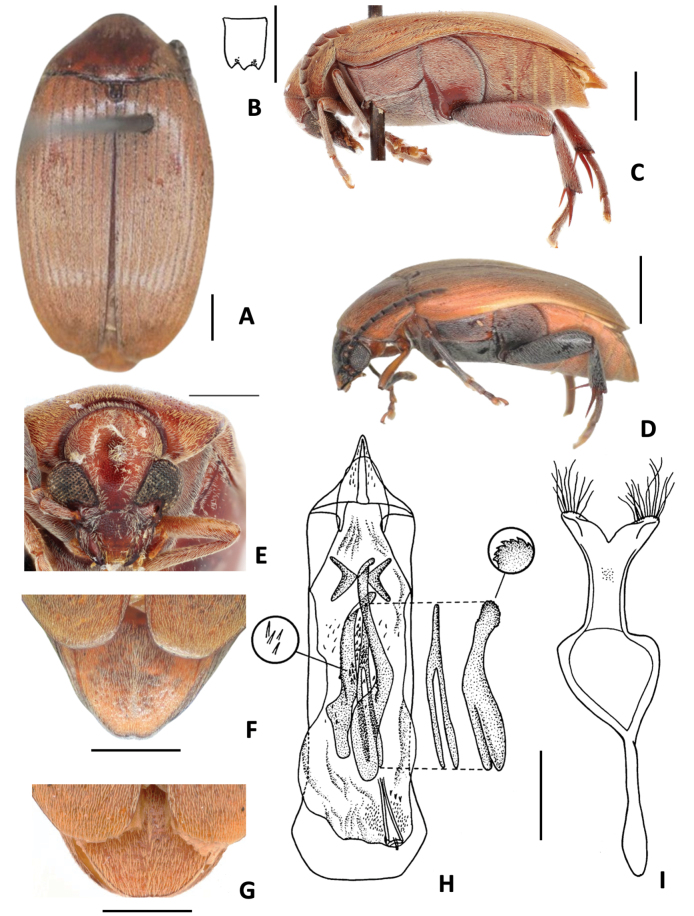
*Amblycerus sclerolobii* Ribeiro-Costa, 2000 **A** dorsal view **B** scutellum **C** lateral view **D** lateral view variation **E** head frontal view **F** male pygidium **G** female pygidium **H** median lobe of male genitalia **I** tegmen of male genitalia. Scale bars: 1.0 mm (**A, C–G**); 0.5 mm (**B, H, I**).

**Figure 7.**
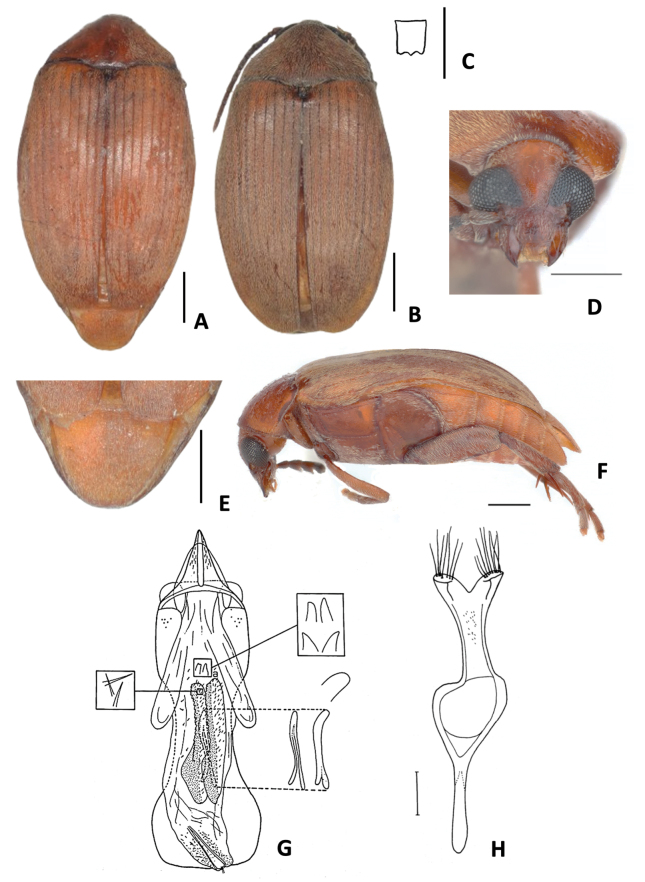
*Amblycerus kingsolveri* Ribeiro-Costa, 1993 **A** dorsal view **B** dorsal view variation **C** scutellum **D** head frontal view **E** male pygidium **F** lateral view **G** median lobe of male genitalia **H** tegmen of male genitalia. Scale bars: 1.0 mm (**A, B–F**); 0.5 mm (**C, G, H**).

**Figure 8.**
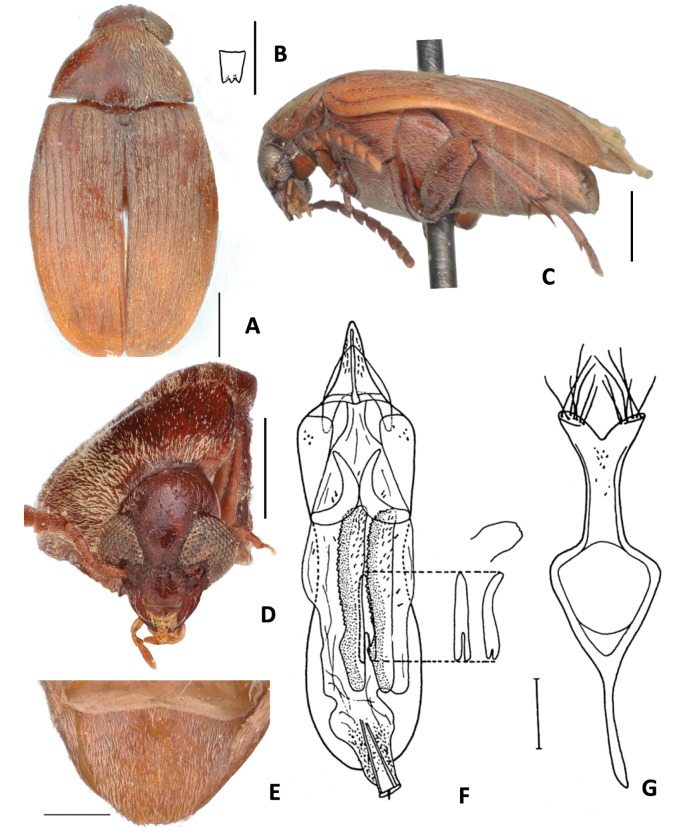
*Amblycerus marinonii* Ribeiro-Costa, 1993 **A** dorsal view **B** scutellum **C** lateral view **D** head frontal view **E** male pygidium **F** median lobe of male genitalia **G** tegmen of male genitalia. Scale bars: 1.0 mm (**A, C, D**); 0.5 mm (**B, E, F, G**).

**Figure 9.**
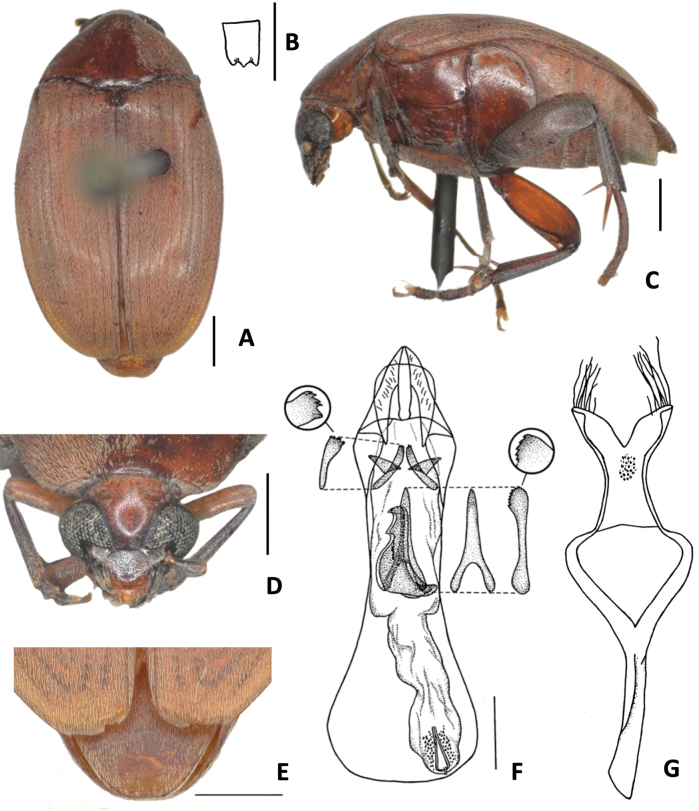
*Amblycerus manauara* Ribeiro-Costa, 2000 **A** dorsal view **B** scutellum **C** lateral view **D** head frontal view **E** male pygidium **F** median lobe of male genitalia **G** tegmen of male genitalia. Scale bars: 1.0 mm (**A, C–E**); 0.5 mm (**B, F, G**).

**Figure 10.**
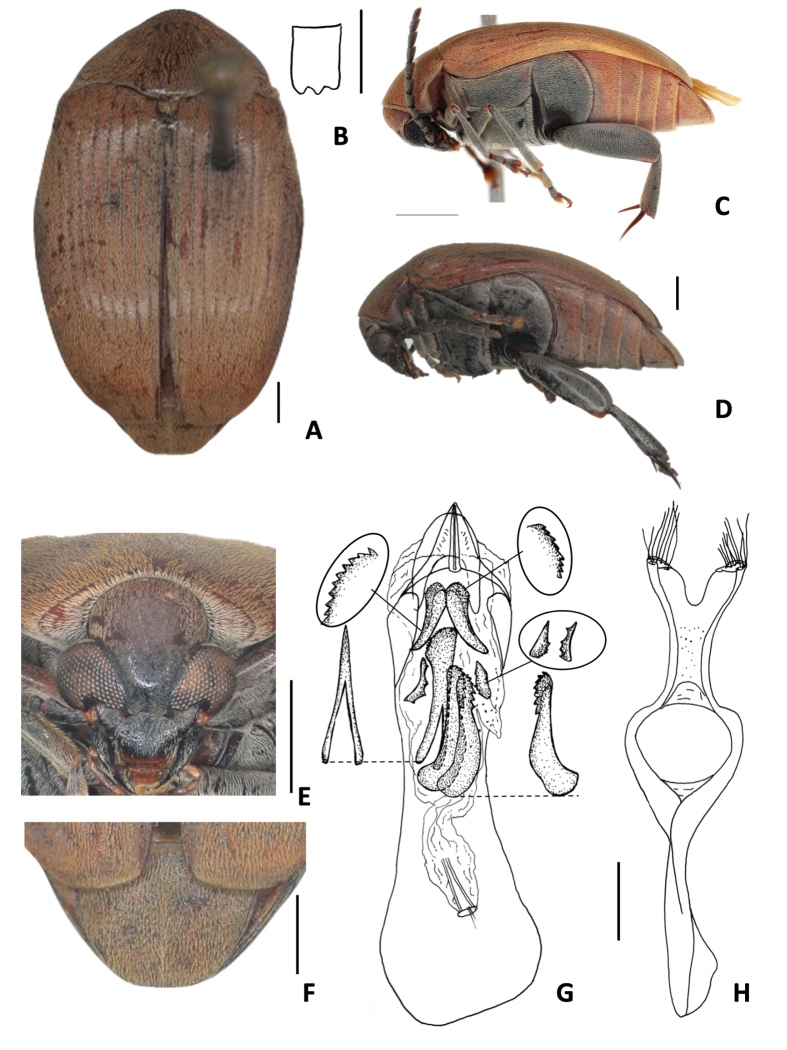
*Amblycerus tachigaliae* Kingsolver, 1976 **A** dorsal view **B** scutellum **C** lateral view **D** lateral view variation **E** head frontal view **F** male pygidium **G** median lobe of male genitalia **H** tegmen of male genitalia. Scale bars: 1.0 mm (**A, C–F**); 0.5 mm (**B, G, H**).

It is difficult to recognize affinities within the *sclerolobii* group. Many of the external characters are variable and the internal characters of the sclerites of the internal sac show different affinities among species. For example, if we consider the laminar serrate sclerites at MR, we will recognize the following relations: winding sclerites shared by *A. falcorostrus* Ribeiro-Costa & Morse, sp. nov. (Fig. 3F), *A. biacutus* Ribeiro-Costa, sp. nov. (Fig. 4F), *A. morsei* Ribeiro-Costa, sp. nov. (Fig. 5G) and *A. sclerolobii* (Fig. 6H); straight sclerites shared by *A. kingsolveri* (Fig. 7G), *A. marinonii* (Fig. 8F) and *A. truncatus* Ribeiro-Costa, sp. nov. (Fig. 2). *Amblycerus manauara* (Fig. 9F) has laminar sclerites with different formats with two teeth each and, in *A. tachigaliae* (Fig. 10G) the laminar sclerite is straight, although the serration is restricted to the subapical area. On the other hand, if we observe the number of sclerites at AR of the internal sac, the affinities among species are: unique sclerite: *A. truncatus* Ribeiro-Costa, sp. nov. (Fig. 2), *A. falcorostrus* Ribeiro-Costa & Morse, sp. nov. (Fig. 3F), *A. biacutus* Ribeiro-Costa, sp. nov. (Fig. 4F), *A. morsei* Ribeiro-Costa, sp. nov. (Fig. 5G); one pair: *A. sclerolobii* (Fig. 6H), *A. tachigaliae* (Fig. 10G), *A. kingsolveri* (Fig. 7G), *A. marinonii* (Fig. 8F); two pairs: *A. manauara* (Fig. 9F). These both male genitalia characters show different internal subgroupings. But, in general, we consider here *A. manauara* and *A. tachigaliae* the most distant species of the group *sclerolobii*.

**Sexual dimorphism.** Pygidium emargination at apex variable between sexes. Last abdominal ventrite of male emarginate at apex in some species.

**Distribution**. Neotropical region: recorded from Panamá to Brazil, from north to southeast region, most commonly found in the Amazon region (Table 1). During the development of the circumscription of the *sclerolobii* group it was possible to allocate one isolated Central American species into this group, *A. tachigaliae*. We also provisionally record here for the first time this species to Brazil (Amazonas-Manaus) including complete data labels based on specimens deposited at TAMU and identified by J. Romero-Nápoles. However, given the description of new closely related species from Brazil, the identifications of these specimens should be confirmed using the distinguishing characters provided in this paper. The other species of the group are exclusively Brazilian, and current records indicate that they are mainly distributed in the northern region of Brazil in the Amazonia Biome (*A. truncatus* Ribeiro-Costa sp. nov., *A. falcorostrus* Ribeiro-Costa & Morse, sp. nov., *A. biacutus* Ribeiro-Costa sp. nov., *A. morsei* Ribeiro-Costa sp. nov., *A. manauara*, *A. sclerolobii* and *A. kingsolveri*). The exception is *A. marinonii* that occurs in the midwest region of Brazil; as its holotype from 1992 and subsequent collecting records from 1998 and 2009 suggest its distribution is clearly in the Cerrado Biome, differing strikingly from the other Brazilian species of the group. *Amblycerus sclerolobii* is the unique Brazilian species with an extended distribution from Northern to Southeast, in the Atlantic Forest.

**Host plants** (Table 1). Fabaceae: *Tachigali denudata* (Vogel) Oliveira-Filho, *Tachigali rubiginosa* (Mart. ex Tul.) Oliveira-Filho, *Tachigali versicolor* Standl. & L.O. Williams, *Dimorphandra macrostachya* Benth, *Dinizia excelsa* Ducke and *Stryphnodendron* sp. (Table 1). All known hosts of species within the species group are rainforest trees in the Caesalpinoideae clade of the Fabaceae (Bruneau et al. 2024), the family that is the preferred host for species of the subfamily Bruchinae (Morse 2014). *Dimorphandra macrostachya*, *Dinizia excelsa*, and the genus *Tachigali* are in the closely related tribes Dimorphandreae, Campsiandreae, and Sclerolobieae, respectively. These three lineages include nine genera with 140–165 species of trees in Neotropical forests (Bruneau et al. 2024), with the genus *Tachigali* making up more than half of this diversity with 80–90 species. Three species of the group, *A. sclerolobii*, *A. marinonii*, and *A. tachigaliae*, share this diverse genus as a host. The genus *Stryphnodendron* is somewhat more distantly related within the tribe Mimoseae; it is a member of the *Stryphnodendron* clade that also includes forest trees in the Neotropics and is composed of seven genera and 56 species (Bruneau et al. 2024).

### ﻿Key to the species of the *sclerolobii* group based on males

**Table:** 

1	Scutellum tridentate, all teeth the same size (Figs 3B, 4B, 5B, 6B, 7C, 8B, 9B, 10B)	**2**
–	Scutellum truncate (Fig. 1B)	***Amblycerus truncatus* Ribeiro-Costa, sp. nov.**
2	Armature of internal sac, AR with a single sclerite or one pair of sclerites (Figs 3F, 4F, 5G, 6H, 7G, 8F, 10G	**3**
–	Armature of internal sac, AR with two pairs of sclerites (Fig. 9F)	***Amblycerus manauara* Ribeiro-Costa, 2000**
3	Armature of internal sac, AR with a single sclerite (Figs 3F, 4F, 5G)	4
–	Armature of internal sac, AR with one pair of sclerites (Figs 6H, 7G, 8F, 10G)	**5**
4	Armature of internal sac, MR with wishbone-shaped sclerite with serrate margin (Fig. 3F)	***Amblycerus falcorostrus* Ribeiro-Costa & Morse, sp. nov.**
–	Armature of internal sac, MR with wishbone-shaped sclerite with smooth apical margin (Figs 4F, 5G)	**6**
5	Armature of internal sac, MR with a pair of winding, long, laminar sclerites (Fig. 6H)	***Amblycerus sclerolobii* Ribeiro-Costa, 2000**
–	Armature of internal sac, MR with a pair of straight sclerites (Figs 7G, 8F, 10G)	**7**
6	Pronotum with lateral carina almost reaching the anterior margin. Armature of internal sac, MR with wishbone-shaped sclerite short, large, rounded at apex (Fig. 4F)	***Amblycerus biacutus* Ribeiro-Costa, sp. nov.**
–	Pronotum with lateral carina reaching ⅔ its length. Armature of internal sac, **MR** with wishbone-shaped sclerite long, slender, acute at apex (Fig. 5G)	***Amblycerus morsei* Ribeiro-Costa, sp. nov.**
7	Armature of internal sac, MR with wishbone-shaped sclerite shorter than the laminar sclerites (Figs 7G, 8F)	**8**
–	Armature of internal sac, MR with wishbone-shaped sclerite longer than the laminar sclerites (Fig. 10G)	***Amblycerus tachigaliae* Kingsolver, 1976**
8	Armature of the internal sac, AR with a spine-shaped pair of sclerites small and straight or gently curved (Fig. 7G)	***Amblycerus kingsolveri* Ribeiro-Costa, 1993**
–	Armature of the internal sac, AR with a tooth-shaped pair of sclerites large and strongly curved (Fig. 8F)	***Amblycerus marinonii* Ribeiro-Costa, 1993**

#### ﻿ Amblycerus truncatus

Taxon classificationAnimaliaColeopteraChrysomelidae

Ribeiro-Costa sp. nov.

1E5DF499-BB5C-5056-8285-F989AEB90B32

https://zoobank.org/2D9F3E39-7DE3-4D91-8C60-C5BF2E373E84

Figs 1A–H, 2A, B; Table 1

##### Type material.

***Holotype*** • Deposited in DZUP with labels: Sinop-MT-Brasil\ 12-14/I/2000\ Caseiro, F.T. col., [white label, printed in black]; Planta hospedeira\ *Dimorphandra macrostachya* [white label, printed in black]; HOLOTYPE\ *Amblycerus truncatus*\ Ribeiro-Costa, [white label with red margin, printed in black]. ***Paratypes*** • (*n* = 5) with the same data as holotype, PARATYPE\ *Amblycerus truncatus*\ Ribeiro-Costa [white label with yellow margin, printed in black]. Two paratypes deposited in DZUP and three paratypes in USDC.

##### Diagnosis.

*Amblycerus truncatus* Ribeiro-Costa, sp. nov. is the unique species in the group *sclerolobii* with truncate scutellum at apex (Fig. 1B) and can easily be identified by this character; the other species in the group have scutellum tridentate, with all teeth the same size. The internal characters of male genitalia also distinguish this species, mainly the unique sclerite at AR of internal sac U-shaped inverted with the stems further apart or only the transverse part of the U (Fig. 2A).

##### Description

. ***Dimensions***: BL: 7.50–8.10 mm (x̄ = 7.86 mm; *n* = 6); BW: 4.70–5.00 mm (x̄ = 4.92 mm; *n* = 6)

***Integument color***: Reddish brown in following areas: first two antennomeres, apex of clypeus, labrum, pronotum, elytra, pygidium, metanepisternum, abdominal ventrites on lateral areas and first, middle legs and hind coxae. Dark brown: part of head, antennomeres except first two, thorax ventrites, abdominal ventrites on middle region and hind leg except coxae. Sometimes specimens entirely darker or paler but in general keep the contrast of colors, at least on antennomeres.

***Vestiture***: Dorsum covered with yellowish pubescence evenly distributed, sometimes whitish on darker integument; pygidium sometimes with faint dense median line of setae.

***Head***: Covered with fine and dense punctures, except clypeus at apex and middle to apex of labrum; punctures gently finer on vertex and coarser on clypeus. Frons convex, frontal carina absent, finely punctate or smooth line on midline, sometimes evanescent basally. Eye coarsely faceted, strongly prominent laterally. Ocular index 4.2; ocular *sinus* 0.1 of the eye length in lateral view; postocular lobe 0.2 the eye length. Antennae with antennomeres elongate except 2 ~ 1/3 the length of 1, moderately serrate from antennomeres 4–10; 11 elongate-acuminate. Frontoclypeal suture conspicuous. ***Prothorax***: Pronotum semicircular, disk moderately convex, with lateral margins moderately arcuate in dorsal view, median basal lobe delimited, not carinate; cervical sulcus on 1/3 lateral reaching cervical boss with trichobothria; disc with fine and dense punctures, moderately coarse punctures on lateral areas; lateral carina almost reaching the anterior margin of pronotum. Prosternal process narrow, apex slightly expanded and gently beyond anterior coxae. ***Mesothorax and metathorax***: Scutellum 1.3 longer than wide with truncate apex. Elytron 2.6 × longer than wide; striae composed of fine punctures joined in lines; all free apically except third and eighth joined; strial intervals only with fine punctures, somewhat flat medially, truncate apically. Mesoventrite truncate apically. Metanepisternum with only fine punctures, lacking striate file; metepisternal sulcus forming right angle, vertical axis not reaching the margin of metepisternum, longitudinal axis less than a half the length of metepisternum. Metaventrite with dense and fine punctures, without coarse punctures, not protuberant between mid coxae, median sulcus extending laterally a half or more than a half the length of metaventrite. Hind coxae with fine dense punctures; moderately coarse, sparse punctures on distal two-thirds. Hind femur slender, ~ 2.82 × longer than wide. Hind tibia with coronal denticles of about equal length; lateral spur ~ 2.36 × the length of median spur. First tarsomere ~ 1.20 the length of lateral spur and 2.84 × the length of median spur. ***Abdomen***: Pygidium basal 1/3 covered by the elytra, with moderately coarse and dense punctures, apex subtruncate in males and subacute in females. Ventrites with moderately coarse, sparse punctures on lateral areas, last ventrite longer than ventrive IV, gently emarginate in male, rounded in female.

***Male terminalia***: Tergite VIII subsquare, subtruncate at apex. Median lobe ~ 3.95 × its widest at apical region; dorsal valve 0.73 × wider than long, subtriangular, lateral margins gently curved, apex rounded; ventral valve 1.59 × wider than long, lateral margins emarginate, apex acute. Armature of internal sac, AR with one sclerite U- shaped inverted with the stems further apart or only the transverse part of the U. MR with a pair of long, laminar, straight sclerites, outer margin serrate along its middle apical 0.5 length; wishbone-shaped sclerite 0.42 as long as the laminar sclerites length, gently curved, apex projected, subtruncate in lateral view or wishbone-shaped sclerite with similar form, less than half its length. BR with one sclerite with long stems gradually approximates. Tegmen with lateral lobes cleft to 1/12 of their length, strongly expanded laterally.

##### Etymology.

The specific name, an adjective in the nominative case, is Latin for truncate in reference to the shape of the scutellum of this species.

##### Distribution.

Brazil (Mato Grosso-Sinop)

##### Host plant.

Fabaceae: *Dimorphandra macrostachya* Benth.

#### ﻿ Amblycerus falcorostrus

Taxon classificationAnimaliaColeopteraChrysomelidae

Ribeiro-Costa & Morse sp. nov.

6DD3AA83-690C-52D7-92A4-70FD203A9B15

https://zoobank.org/64C48323-ECFB-4A1C-B9A7-91D92745CE4F

Fig. 3A–G, Table 1

##### Type material.

***Holotype*** • Deposited in INPA, male, with labels: Brasil: Amazonas\ AM 010, Km 26\ Reserva Ducke\ 29-VIII-1978 [white label, printed in black except date handwritten]; armadilha\ de Malaise [white label, printed in black]; INPA [white label with black margin, printed in black]; Amblycerus\ sp.2\ Ribeiro-Costa, C.S. det. 2009 [white label with black margin, handwritten except name of identifier and date printed in black]; HOLOTYPE\ *Amblycerus falcorostrus*\ Ribeiro-Costa & Morse [white label with red margin, printed in black].

##### Diagnosis.

The main character to distinguish *A. falcorostrus* Ribeiro-Costa & Morse, sp. nov. from others in the group is the presence in the internal sac of male genitalia of a single, strongly curved tooth-shaped sclerite on both sides at AR (Fig. 3F).

##### Description.

The description of *A. falcorostrus* Ribeiro-Costa & Morse, sp. nov. is similar to *A. truncatus* Ribeiro-Costa, sp. nov. except by the following characters.

***Dimensions***: BL: 6.60 mm, BW: 3.80 mm (*n* = 1).

***Integument color***: Dark brown in following areas: vertex, apex of clypeus, labrum, pronotum, elytra, pygidium, abdominal ventrites, first femur and middle leg. Black on part of head, antennomeres, metanepisternum, metasternum, and hind leg.

***Vestiture***: Ventral region with whitish pubescence except abdominal ventrites with yellowish pubescence.

***Head***: Frontal carina absent, finely punctate or smooth on midline. Ocular index: 5.3; ocular sinus 0.05 of the eye length in lateral view. ***Prothorax***: Pronotum with carinae bordering the basal lobe and laterally. ***Mesothorax and metathorax***: Scutellum about as long as wide with tridentate apex, all teeth the same size. Elytron 1.4 × longer than wide. Metanepisteral sulcus with the vertical axis reaching the margin of metepisternum. Metaventrite with median sulcus extending laterally more than a half of its length. Hind femur ~ 2.72 × longer than wide. Hind tibia lateral spur ~ 2 × the length of median spur. First tarsomere ~ 1.38 × the length of lateral spur and 2.61 × the length of median spur. ***Abdomen***: Pygidium rounded at apex in male, female unknown. Last ventrite almost the same length as ventrite IV, male apex not visible. ***Male terminalia***: Tergite 8 gently emarginate at apex. Median lobe ~ 5.34 × its widest at apical region; dorsal valve 0.88 × wider than long; ventral valve 1.28 × wider than long. Armature of internal sac, AR with one strongly curved tooth-shaped sclerite in both sides. MR with a pair of winding, laminar sclerites, outer margin serrate along its middle apical 0.7 length; wishbone-shaped sclerite 0.63 as long as the laminar sclerites length, straight, apex enlarged, subtruncate and serrate in lateral view. Tegmen with lateral lobes cleft 1/16 of their length.

##### Etymology.

The specific name is a new Latin adjective in the nominative case meaning “with a falcon-like beak”. It refers to the form of the AR sclerite in the internal sac of the male genitalia.

##### Distribution.

Brazil (Amazonas-Manaus).

##### Host plants.

Not known.

#### ﻿ Amblycerus biacutus

Taxon classificationAnimaliaColeopteraChrysomelidae

Ribeiro-Costa sp. nov.

E932F33B-BA9D-5478-B9AC-19CE62D3923D

https://zoobank.org/CB3488FE-10E9-457A-8388-45938AEE0A41

Fig. 4A–G, Table 1

##### Type material.

***Holotype*** • Deposited in USNM, male, with labels: Ex Stryphnodendron\ SP. Krukoff\ 800S – Brazil, AM.\ Manaus, IX/X.1934 [white label, handwritten in black]; extracted from\ herbarium\ specimen [white label, printed in blue]; Amblycerus prov. nova prox.\ A. sclerolobii \C.S. Ribeiro-Costa det. 199.., [white label with black margin, handwritten except name of identifier and date printed in black]; HOLOTYPE\ *Amblycerus biacutus*\ Ribeiro-Costa [white label with red margin, printed in black].

##### Diagnosis.

This species can be separated from the others by the internal sac of male genitalia with a single, gently curved on both sides and acute at each apex (Fig. 4F). It can never be confused with *A. falcorostrus* Ribeiro-Costa & Morse, sp. nov. if comparing this sclerite at AR because of the curvature at apex: in *A. biacutus* Ribeiro-Costa, sp. nov., it is gently curved; in *A. falcorostrus* Ribeiro-Costa & Morse, sp. nov. it is strongly curved. Besides, differences can be found in the length at the apex and form of the wishbone-shaped sclerite at RM: in *A. biacutus* Ribeiro-Costa, sp. nov., it is short and the apex smooth; in *A. falcorostrus* Ribeiro-Costa & Morse, sp. nov. it is long and the apex serrated (Fig. 3F).

##### Description.

The description of *A. biacutus* Ribeiro-Costa, sp. nov. is similar to *A. truncatus* Ribeiro-Costa, sp. nov. except in the following characters.

***Dimensions***: BL: 6.70 mm, BW: 4.00 mm (*n* = 1).

***Integument color***: Similar to paler specimens of *A. truncatus* Ribeiro-Costa, sp. nov., except all antennomeres dark brown. ***Vestiture***: Ventral region with whitish pubescence except abdominal ventrites with yellowish pubescence. ***Head***: Frontal carina faint basally. Ocular index 3.94; ocular *sinus* 0.2 of the eye length in lateral view. ***Prothorax***: Pronotum with carina bordering the basal lobe. ***Mesothorax and metathorax***: Scutellum ~ 1.5 longer than wide with tridentate apex, all teeth the same size. Elytron 2.85 × longer than wide. Metanepisternal sulcus forming right to gentle obtuse angle, vertical axis reaching the margin of metepisternum. Metaventrite with median sulcus extending > 1/2 of its length. Hind femur ~ 2.45 × longer than wide. Hind tibia, lateral spur ~ 2 × length of median spur. First tarsomere ~ 1.38 × the length of lateral spur and 2.76 × the length of median spur. ***Abdomen***: Pygidium with truncate apex in male, female unknown. Last ventrite almost the same length as ventrite IV, apex truncate in males, females unknown. ***Male terminalia*.** Tergite VIII gently emarginate at apex. Median lobe ~ 4.20 × its widest at apical region; dorsal valve 0.75 × wider than long, lateral margins straight, apex subtruncate; ventral valve 1.18 × wider than long. Armature of internal sac, AR with a single, gently curved sclerite on both sides and, acute at each apex. MR with a pair of winding, laminar sclerites, outer margin serrate along its middle apical 0.5 of length; wishbone-shaped sclerite 0.63 as long as the laminar sclerites length, straight, apex gently curved and smooth in lateral view. Tegmen with lateral lobes cleft to 1/11 of their length.

##### Etymology.

The specific name, an adjective in the nominative case, is Latin for “twice acute”. It refers to the apex of the AR sclerite in the internal sac of male genitalia.

##### Distribution.

Brazil (Amazonas-Manaus).

##### Host plant.

Fabaceae: *Stryphnodendron* sp.

#### ﻿ Amblycerus morsei

Taxon classificationAnimaliaColeopteraChrysomelidae

Ribeiro-Costa sp. nov.

FF8B1728-FE5A-5B7D-AEA2-B755A5FAE490

https://zoobank.org/83E991C8-57CF-49B1-B7D2-F537854B55F1

Fig. 5A–H, Table 1

##### Type material.

***Holotype*** • Deposited in DZUP, male, with labels: Brasil– AM, 80 Km N\ Manaus VI–VIII 1996\ 2°30'S, 60°00'W\ C.W. Dick col. [white label, printed in black]; Larva consumindo semente de Dinizia excelsa [white label, printed in black], Biological Dynamics of\ Forest Fragments Project\ (BDFFP) [white label, printed in black]. HOLOTYPE\ *Amblycerus morsei*\ Ribeiro-Costa [white label with red margin, printed in black]. ***Paratypes*** • (*n* = 19), same data as holotype, PARATYPE\ *Amblycerus morsei*\ Ribeiro-Costa [white label with yellow margin, printed in black]. Six deposited in DZUP, two in MZSP, two in MNRJ, three in USNM, and six in SDMC.

##### Diagnosis.

The main character to distinguish *Amblycerus morsei* Ribeiro-Costa, sp. nov. from others of the group is the unique strongly curved tooth-shaped sclerite at AR (Fig. 5G). *Amblycerus falcorostrus* Ribeiro-Costa & Morse, sp. nov. and *A. biacutus* Ribeiro-Costa, sp. nov. share a unique sclerite at AR but their sclerites have projections for both sides (Figs 3F, 4F). However, in *A. kingsolveri*, *A. marinonii*, *A. sclerolobii*, and *A. tachigaliae* have one pair of sclerites (Figs 6H, 7G, 8F, 10G) and *A. manauara* has two pairs (Fig. 9F).

##### Description.

The description of *A. morsei* Ribeiro-Costa, sp. nov. is similar to *A. truncatus* Ribeiro-Costa, sp. nov. except by the following characters.

***Dimensions***: BL: 5.50–6.50 mm (x̄ = 5.89 mm; *n* = 10); BW: 3.30–3.70 mm (x̄ = 3.49 mm; *n* = 10).

***Integument color***: Similar to color variations in *A. truncatus* Ribeiro-Costa, sp. nov., some specimens darker or paler but in general keeping the contrast of colors. Antenna with the first two antennomeres reddish brown, the rest dark brown; sometimes entirely dark brown.

***Vestiture***: Dorsum covered with yellowish pubescence evenly distributed, sometimes near elytral suture with whitish setae. Ventral region with whitish pubescence except abdominal ventrites with yellowish pubescence.

***Head***: Frontal carina absent, finely punctate or sometimes less punctate on midline. Ocular index 4.54; ocular sinus 0.02 of the eye length in lateral view; postocular lobe 0.03 of the eye length. ***Prothorax***: Pronotum with carina bordering the basal lobe and toward part of laterals; lateral carina ⅔ the length of pronotum. ***Mesothorax and metathorax***: Scutellum ~ 1.5 × longer than wide with tridentate apex, all teeth the same size. Elytron 1.43 × longer than wide, subtruncate apically. Metanepisternal sulcus with the vertical axis reaching the margin of metepisternum, longitudinal axis ~ less or half the length of metepisternum. Hind femur ~ 2.76 × longer than wide. Hind tibia, lateral spur ~ 1.86 × the length of median spur. First tarsomere ~ 1.58 × the length of lateral spur and 2.93 × the length of median spur. Pygidium 1/3 or 1/2 covered by the elytra. ***Abdomen***: Pygidium at apex truncate in males, emarginate in females. Last ventrite longer than ventrite IV, apex without differences between sexes. ***Male genitalia***: Median lobe ~ 3.60 × its widest at apical region; dorsal valve 1.30 × wider than long, subtriangular, lateral margins almost straight, apex rounded ventral valve 1.3 × wider than long, lateral margins almost straight, apex acute; dorsal valve 0.73 × wider than long, subtriangular, lateral margins curved; ventral valve 1.59 × wider than long. Armature of internal sac, AR with one strongly curved tooth-shaped sclerite. MR with a pair of winding, laminar sclerites, outer margin serrate along its middle apical 1/2 length; wishbone-shaped sclerite 0.49 × as long as the laminar sclerites length, straight, apex enlarged and serrated in lateral view. Tegmen with lateral lobes cleft to 1/19 of their length.

##### Etymology.

The specific name is named in honor of Geoffrey E. Morse for his enthusiasm, dedication, and competence to the study of seed beetles.

##### Distribution.

Brazil (Amazonas-Manaus).

##### Host plant.

Fabaceae: *Dinizia excelsa* Ducke.

#### ﻿ Amblycerus sclerolobii

Taxon classificationAnimaliaColeopteraChrysomelidae

Ribeiro-Costa, 2000

8B57DDB7-4C4D-5EB7-9ABC-6F57DCB62B0C

Fig. 6A–I, Table 1

Amblycerus sclerolobii Ribeiro-Costa, 2000: 328–330 (detailed original description, type locality: Amazonas, Minas Gerais); Ribeiro-Costa et al. 2018: 515 (catalog); Santos and Ribeiro-Costa 2019: 103 (taxonomy); Romero-Nápoles et al. 2021: 209 (catalog).

##### Type material.

***Holotype*** • Deposited in DZUP, male, with labels: Brasil: Minas Gerais, Viçosa; 19-XI-986; G.P.Santos *leg.*, em semente de mamoneira-branca, *Sclerolobium* sp. ***Paratypes*** • (*n* = 11), eight with the same data as holotype, two paratypes deposited in DZUP, two in MZSP, two in MNRJ, two in USNM • one Brasil: *Amazonas*, Rio Javari, Estirão do Ecuador; X.1979; M .Alvarenga *leg.*, in CMNH (Carnegie Museum of Natural History, Oakland, Pennsylvaniaand) and • two only with the information 5.X.1936, (EQ 045238), *Sclerolobium denudatum*, in USNM (Ribeiro-Costa 2000).

##### Diagnosis.

*Amblycerus sclerolobii* differs from the others of the group by the armature of the internal sac of male genitalia. The AR has one pair of sclerites (Fig. 6H) (*A. truncatus* Ribeiro-Costa, sp. nov., *A. falcorostrus* Ribeiro-Costa & Morse, sp. nov., *A. biacutus* Ribeiro-Costa, sp. nov., *A. morsei* Ribeiro-Costa, sp. nov. has one sclerite- Figs 2, 3F, 4F, 5G; *A. manauara* has two pairs- Fig. 9F), but *A. marinonii*, *A. kingsolveri* and *A. tachigaliae* share with *A. sclerolobii* one pair (Figs 7G, 8F, 10G). Although *A. sclerolobii* can be easily separated from these last three species by the serrate winding laminar sclerites at MR (Fig. 6H) (*A. kingsolveri*, *A. marinonii*, *A. tachigaliae* have straight laminar sclerites: Figs 7G, 8F, 10G).

##### Note.

Ribeiro-Costa (2000) described this species as having the general integument color contrasting with the antennae, thoracic ventrites, and legs. Here we present illustrations of the extremes of this contrast (Fig. 6C, D).

##### Distribution.

Brazil (Amazonas-Estirão do Equador, Minas Gerais-Viçosa).

##### Host plants.

Fabaceae: *Tachigali denudata* (Vogel) Oliveira-Filho, *Tachigali* sp. (quoted as *Sclerolobium denudatum*, *Sclerolobium* sp.).

#### ﻿ Amblycerus kingsolveri

Taxon classificationAnimaliaColeopteraChrysomelidae

Ribeiro-Costa, 1993

C0A4496D-9EDB-56F8-A117-4A1D23285D6D

Fig. 7A–H, Table 1

Amblycerus kingsolveri Ribeiro-Costa, 1993: 5–8 (detailed original description, type locality: Amazonas); Ribeiro-Costa 2000: 330 (mentioned); Ribeiro-Costa et al. 2018: 509 (catalog); Santos and Ribeiro-Costa 2019: 103 (taxonomy); Romero-Nápoles et al. 2021: 208 (catalog).

##### Type material.

***Holotype*** • Deposited in DZUP, male, with labes: Brasil: Amazonas, Rio Tarumã Mirim, 2Km from Rio Negro, 03°02'S-60°17'W; 30 July 79; Adis, Erwin, Montgomery leg. ***Paratype*** • (*n* = 1), male, Brazil: Amazonas, Manaus, 1941 in DZUP. (Ribeiro-Costa 1993).

##### Additional material examined.

• [*n* = 2] Brasil: Amazonas\ Rio Tarumã Mirim\ 20 KM nw Manaus\ 02Mar1979/ 02°53'S, 060°07'W [white label, printed in black]; Montgomery, Erwin,\Schimmel, Krishik, \Date, Bacon Colls [white label, printed in black]; Black water innun-\dation forest canopy\ fogged with pyrethrum\ Sample #39 [white label, printed in black]; Amblycerus\ kingsolveri\ Ribeiro-Costa, C. S. det. 2024 [white label with black margin, handwritten except name of identifier and date printed in black] (USNM).

##### Diagnosis.

This species is easily recognized by the unique small pair of spine-shaped sclerite at AR of the internal sac of the male (Fig. 7G). The other species in the *sclerolobii* group have large sclerites with different forms.

##### Note.

Ribeiro-Costa (1993) described this species as having the general integument color reddish yellow as in Fig. 7A and here we present an illustration of the darker specimens (Fig. 7B).

##### Distribution.

Brazil (Amazonas-Manaus, Rio Tarumã Mirim).

##### Host plant.

Unknown.

#### ﻿ Amblycerus marinonii

Taxon classificationAnimaliaColeopteraChrysomelidae

Ribeiro-Costa, 1993

787A4E53-63E5-56C9-8E28-43CC413D98DD

Fig. 8A–G, Table 1

Amblycerus marinonii Ribeiro-Costa, 1993: 8–9 (detailed original description, type locality: Goiás); Ribeiro-Costa 2000: 330 (mentioned); Ribeiro-Costa et al. 2018: 551 (catalog); Santos and Ribeiro-Costa 2019: 103 (taxonomy); Romero-Nápoles et al. 2021: 208 (catalog).

##### Type material.

***Holotype*** • Deposited in DZUP, male, with labels: Brazil: Goiás, Dianópolis; 16-22.I.1962; J. Bechyné leg. (Ribeiro-Costa 1993).

##### Additional material examined.

• (*n* = 5) Cuiabá-MT/X/1998\Souza, M.J.M.\pl.hosp.\Sclerolobium\ paniculatum\ var. rubiginosum[white label with black margin, printed in black]; Amblycerus\ marinonii\ Ribeiro-Costa, C. S. det. 2024 [white label with black margin, handwritten except name of identifier and date printed in black] (USNM). **New record**. • (*n* = 2) Brazil – Go Barro Alto\ 15°04'23.30"S, 48°59'51.60"W \XI 2009 M. Pimenta & L.L.\ Bergamini cols Malaise[white label with black margin, handwritten except name of identifier and date printed in black]; Chrysomelidae\Bruchinae sp.4 p831\L.L. Bergamini det. 2010 [white label with black margin, printed in black]; Amblycerus\ marinonii\ Ribeiro-Costa, C. S. det. 2024 [white label with black margin, handwritten except name of identifier and date printed in black] (DZUP).

##### Diagnosis.

*Amblycerus marinonii* can be distinguished mainly by the presence in the internal sac of male genitalia at AR of one pair of tooth-shaped, large and strongly curved sclerite (Fig. 8F). Although *A. marinonii* shares one pair of sclerite with *A. sclerolobii* (Fig. 6H), *A. tachigaliae* (Fig. 10G), *A. marinonii* (Fig. 8F) and *A. kingsolveri* (Fig. 7G) it can be separated by the conspicuous form of the AR sclerites.

##### Distribution.

Brazil (Mato Grosso-Cuiabá, Goiás-Dianópolis, Barro Alto*).

##### Host plants.

Fabaceae: *Tachigali rubiginosa* (Mart. ex Tul.) Oliveira-Filho (quoted as Sclerolobium paniculatum var. rubiginosum).

#### ﻿ Amblycerus manauara

Taxon classificationAnimaliaColeopteraChrysomelidae

Ribeiro-Costa, 2000

B11C780E-9F2E-59E3-BECB-D1471E000272

Fig. 9A–G, Table 1

Amblycerus manauara Ribeiro-Costa, 2000: 330–332 (detailed original description, type locality: Amazonas), Ribeiro-Costa 2000: 330,331 (mentioned); Ribeiro-Costa et al. 2018: 551 (catalog); Santos and Ribeiro-Costa 2019: 103 (taxonomy); Romero-Nápoles et al. 2021: 208 (catalog).

##### Type material.

***Holotype*** • Deposited in USNM, male, with labels: Brasil: *Amazonas*: [Uypizanga], Rio Negro, (14 km de Manaus, Alt. 300 pés); XII. 1941; A. Rabaut leg. (Ribeiro-Costa 2000).

##### Diagnosis.

One of the easiest species to identify in the group *sclerolobii* is *Amblycerus manauara*. It has the most conspicuous character in the male genitalia, the two pairs of sclerites at the AR in the male genitalia (Fig. 9F) which separates this species from the others that have one pair (*A. kingsolveri*, *A. marinonii*, *A. sclerolobii*, *A. tachigaliae*; Figs 7G, 8F, 6H, 10G) or just a single sclerite (*A. truncatus* Ribeiro-Costa, sp. nov., *A. falcorostrus* Ribeiro-Costa & Morse, sp. nov., *A. biacutus* Ribeiro-Costa, sp. nov. and *A. morsei* Ribeiro-Costa, sp. nov.; Figs 2A, 3F, 4F, 5G).

##### Distribution.

Brazil (Amazonas-Manaus).

##### Host plants.

Unknown.

#### ﻿ Amblycerus tachigaliae

Taxon classificationAnimaliaColeopteraChrysomelidae

Kingsolver, 1976

B59D5F85-E189-5FF0-98C1-AD387EDB83D2

Fig. 10A–H, Table 1

Amblycerus tachigaliae Kingsolver, 1976: 150–151 (detailed original description, type locality: Panama); Kingsolver 1980: 238–239 (mentioned); Johnson and Kingsolver 1981: 410 (checklist); Kitajima and Augspurger 1989: 1105 (distribution, host association, ecology); Udayagiri and Wadhi 1989: 15 (catalog); Romero-Nápoles et al. 2021: 210 (catalog).

##### Type material.

***Holotype*** • Deposited in USNM, male, with labels: Panama: Barro Colorado I.; feb. 1975; Robin Foster, coll., reared from seeds of *Tachigali versicolor* Standley & Williams. ***Paratypes*** • (*n* = 2), two same locality, one female; 24-II-1975; T. L. Erwin coll., at light in USNM, one male; 10-III-1961; J. M. Campbell coll., at light, in CNC (Canadian National Collection, Ottawa). (Kingsolver 1976).

This species was recognized based on the original detailed description presented by Kingsolver (1976) where the male genitalia is well illustrated. **New record**. • (*n* = 2) Panama: Colón, Santa Rita Ridge 10–11 June 1977, coll. Howden, H. and Howden • (*n* = 3) Manaus, Brazil\1978 [white label, handwritten]; ♀; collected by\ M.A.S. Serrano [white label, first line printed in black, second handwritten]; Amblycerus tachigaliae\ Kingsolver\ Det. J. Romero N. 91’ (white label, printed in black, first line bold letters); TAMU-ENTO X0102475 (TAMU) [just one with a female label]. As discussed previously, this should be considered provisional and should be confirmed based on the characters presented in the key.

##### Diagnosis.

*Amblycerus tachigaliae* has a number of sclerites in the internal sac of male genitalia that distinguish it from the other species of the group *sclerolobii* (Fig. 10G). It has an extra pair of sclerites at MR, short and denticulate, in addition to the pair of laminar sclerites and unpaired wishbone-shaped sclerites shared by the other species of the group.

##### Note.

In the original description of *A. tachigaliae*, Kingsolver (1976) did not mention the pair of sclerites, short and denticulate at MR (Fig. 10G). However, we can identify them in the drawing of male genitalia of Kingsolver (1976) by carefully observing the teeth placed at the same position as these sclerites. We illustrate the extreme contrasts observed in the integument coloration between ventrites of the thorax, legs, and abdomen for *A. tachigaliae* (Fig. 10C, D).

##### Distribution.

Panama (Barro Colorado Island, Colón*), Brazil (Amazonas-Manaus*)

##### Host plant.

Fabaceae: *Tachigali versicolor* Standl. & L.O. Williams (quoted erroneously as *Tachigalia versicolor*)

###### ﻿Species excluded from the *sclerolobii* group

#### ﻿ Amblycerus bicolor

Taxon classificationAnimaliaColeopteraChrysomelidae

(Pic, 1927)

2D639382-08B6-5BB0-8A8C-012A0708F8B0

Fig. 11A–H, Table 1

Spermophagus bicolor Pic, 1927: 187(original description, distribution).
Amblycerus bicolor : Ribeiro-Costa 1992: 167–168; 172 (redescription, type material, male genitalia); Ribeiro-Costa et al. 2018: 502–503 (catalog); Romero-Nápoles et al. 2021: 207 (catalog).

##### Type material.

This species was redescribed by Ribeiro-Costa (1992). The single ***syntype*** deposited in MNHN (Muséum national d’Histoire naturelle, Paris, France), a female, was examined, with the label • “Itaituba, Amazones”; however, we emphasize here that the locality “Itaituba” is in the Para state of Brazil.

##### Diagnosis.

**Figure 11.**
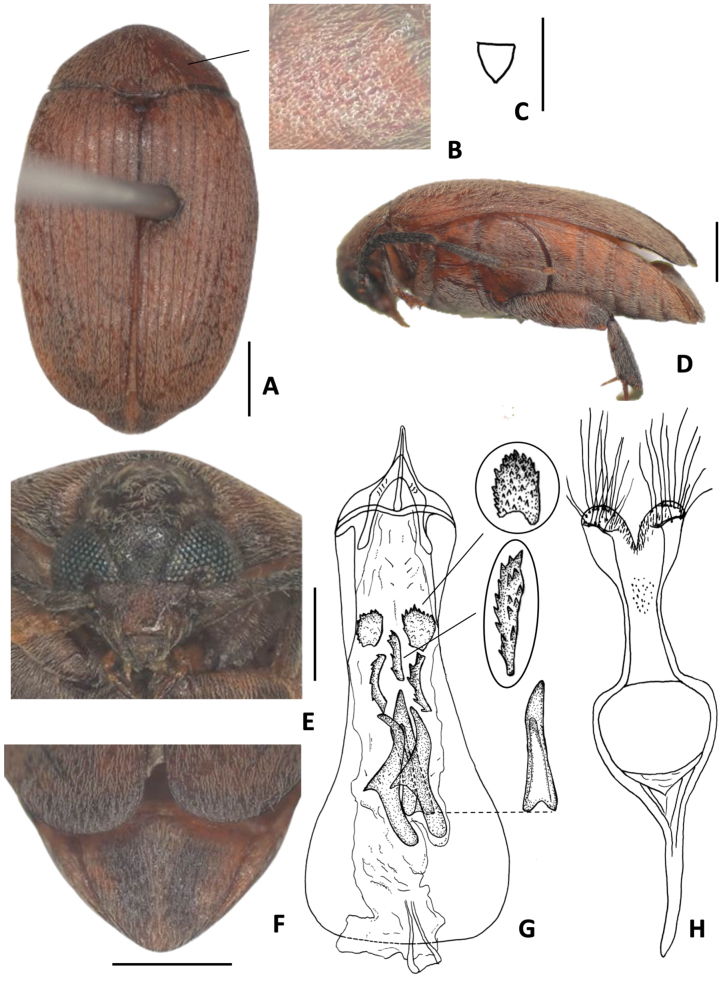
*Amblycerus bicolor* (Pic, 1927) **A** dorsal view **B** punctures on lateral areas of pronotum **C** scutellum **D** lateral view **E** head frontal view **F** male pygidium **G** median lobe of male genitalia **H** tegmen of male genitalia. Scale bars: 1.0 mm (**A, B, D–F**); 0.5 mm (**C, G, H**).

*Amblycerus bicolor* can be distinguished by the species of the *sclerolobii* group and the other *Amblycerus* species by the scutellum with subacute apex (Fig. 11C), pronotum with elongate coarse punctures on lateral areas (Fig. 11B), prosternal process very narrow, laminar; elytra and pygidium with coarse punctures at apex and the color pattern of integument on the pygidium with two darker oval areas (Fig. 11F). It is worth noting that in the male genitalia the two unpaired sclerites at MR differ, one anterior with denticles and the posterior is long, entire, not wishbone-shaped with separate stems (Fig. 11G).

##### Distribution.

Brazil (Para), Suriname, French Guiana.

##### Host plants.

*Cassia* sp. (Fabaceae).

###### ﻿Revision of *Amblycerus* species groups

Table 2

**Table 2.** List of published *Amblycerus* species groups with their composition. Note that there is disagreement between authors, but that all published groups other than those reconciled in this paper are included. Abbreviations for distribution: (W) West Indies, (N) North America north of Mexico, (M) Mexico, (C) Central America, (S) South America. (*) species not included in the analysis of Ribeiro-Costa (1995) but placed in the group.

*Amblycerus* groups	Species composition	Distribution	Authors of groups
* anosignatus *	*A. anosignatus*, *A. chiapas*, *A. guerrerensis*	C, M, S	Romero-Nápoles et al. (1996)
* canescens *	*A. canescens*, *A. teutoniensis*	S, M	Ribeiro-Costa (1995)
* cistelinus *	*A. atypicus*, *A. cistelinus*, A. guazumicola*, *A. jatayensis*, *A. sosia*, A. whiteheadi*	W, N, M, C, S	Ribeiro-Costa (1995)
* championi *	*A. championi*, *A. evangelinae*, *A. teutoniensis*	W, M, C, S	Romero-Nápoles et al. (1996)
* chapadicola *	*A. chapadicola*, *A. germaini*, *A. mourei*	S	Ribeiro-Costa (1995)
* danunciae *	*A. danunciae*	S	Ribeiro-Costa (1995)
* decoris *	*A. decoris*	S	Ribeiro-Costa (1995)
* dispar *	*A. crassipunctatus*, *A. dispar*, *A. insuturatus*, *A. schwarzi*, *A. taeniopygus*, *A. goianensis*	W, N, M, C, S	Ribeiro-Costa (1995)
* epsilon *	*A. epsilon*	M, C, S	Romero-Nápoles et al. (1996)
* geminatus *	*A. geminatus*, A. multiflocculus*, *A. similaris*, *A. similis*	M, C, S	Ribeiro-Costa (1995)
* gounellei *	*A. gounellei*	S	Ribeiro-Costa (1995)
* hoffmanseggi *	*A. hoffmanseggi*, *A. submaculatus*, *A. nigromarginatus*, *A. obscurus*	N, M, C, S	Ribeiro-Costa and Marinoni (1992)
* ischiodontus *	*A. ischiodontus*	S	Ribeiro-Costa (1995)
* isocalcaratus *	*A. isocalcaratus*	S	Ribeiro-Costa (1995)
* luteonotatus *	*A. luteonotatus*	S	Ribeiro-Costa (1995)
* luteolineatus *	*A. luteolineatus*	S	Ribeiro-Costa (1995)
* marmoratus *	*A. marmoratus*, *A. barcenae*, *A. hespenheidei*, *A. pictus*	M, C	Romero-Nápoles et al. (1996)
* megalobus *	*A. atrogaster*, A. championi* , *A. megalobus*, *A. multimaculatus*, *A. nigronotatus*	M, C, S	Ribeiro-Costa (1995)
* paulonotatus *	*A. paulonotatus*, *A. isabelae*, A. spectabilis*	C, S	Ribeiro-Costa (1995)
* perfectus *	*A. bidentatus*, *A. denticulatus*, *A. perfectus*, *A. imperfectus*	M, C, S	Ribeiro-Costa (1995)
* piurae *	*A. piurae*	M, S; introduced into French Polynesia	Romero-Nápoles et al. (1996)
* pollens *	*A. pollens*, *A. spiniger*	C, S	Ribeiro-Costa (1995)
* pterocarpae *	*A. pterocarpae*, *A. luciae*	M, C, S	Ribeiro-Costa (1995)
* profaupar *	A. baracoensis*, A. caymanensis*, *A. cerdanicola*, *A. longesuturalis*, *A. maculicollis*, *A. profaupar*, *A. pusillus*, A. scutellaris*	W, N, M, C, S	Ribeiro-Costa (1995)
* reticulatus *	*A. reticulatus*	S	Ribeiro-Costa (1995)
* robiniae *	*A. acapulcensis*, *A. robiniae*, *A. taeniatus*	W, N, M	Kingsolver (1975)
* robiniae *	*A. acapulcensis*, *A. ireriae*, *A. robiniae*	W, N, M	Romero-Nápoles et al. (1996)
* sclerolobii *	*A. sclerolobii*, *A. kingsolveri*, *A. marinonii*, *A. manauara*, *A. tachigaliae*, *A. biacutus* sp. nov., *A. falcorostrus* sp. nov., *A. morsei* sp. nov., *A. truncatus* sp. nov.	C, S	Here proposed
* scutellaris *	*A. atkinsoni*, *A. baracoensis*, *A. biolleyi*, *A. cerdanicola*, *A. mariae*, *A. montalvoi*, *A. pygidialis*, *A. scutellaris*, *A. veracruz*	W, N, M, C, S	Romero-Nápoles et al. (1996)
* spondiae *	*A. alternatus*, *A. cuernavacensis*, *A. spondiae*, *A. vitis*	N, M, C, S	Romero-Nápoles et al. (1996)
* stridulator *	*A. stridulator*	M, C, S	Romero-Nápoles et al. (1996)
* virens *	*A. medialis*, *A. virens*, *A. virescens*, *A. viridans*, *A. viridis*	S	Ribeiro-Costa et al. (1995)

After recording the *Amblycerus* species placed in groups by different authors (Kingsolver 1975; Ribeiro-Costa 1995; Ribeiro-Costa and Marinoni 1992; Romero-Nápoles et al. 1996) it was possible to note groups with similar names, and in part with similar species or groups with different names and composed in part or entirely by similar species. Therefore similarities among their species composition justify merging groups. The groups below are merged, keeping the senior name of the group.

1. *cistelinus* group (Ribeiro-Costa 1995): *A. cistelinus* (Gyllenhal, 1833), *A. guazumicola* Kingsolver & Johnson, 1971, *A. jatayensis* (Pic, 1902), *A. sosia* Ribeiro-Costa & Kingsolver, 1993a and *A. whiteheadi* Kingsolver, 1991. Romero-Nápoles et al. (1996) subsequently proposed the group with the same name composed by the shared species *A. cistelinus*, *A. guazumicola* and *A. sosia*. Ribeiro-Costa (1999a) described *A. atypicus*, Ribeiro-Costa, 1999a and suggested this species probably belonged to this group, confirmed here. Therefore the *cistelinus* group is composed of six species.
2. *hoffmanseggi* group (Ribeiro-Costa and Marinoni 1992): *A. hoffmanseggi* (Gyllenhal, 1833), *A. submaculatus* (Pic, 1927), *A. nigromarginatus* (Motschulsky, 1874), and *A. obscurus* (Sharp, 1885). Romero-Nápoles et al. (1996) subsequently proposed the group *obscurus* composed of the shared species *A. nigromarginatus* and *A. obscurus*: this group is therefore replaced by the previous group name *hoffmanseggi*.
3. *geminatus* group (Ribeiro-Costa 1995): *A. geminatus* (Sharp, 1885), *A. multiflocculus* Kingsolver, 1980, *A. similaris* Ribeiro-Costa, 1999b, and *A. similis* Ribeiro-Costa, 1999b. Romero-Nápoles et al. (1996) subsequently proposed the group *multiflocculus* composed only of *A. multiflocculus*, here we merge both these groups and keep the name *geminatus* due to seniority.
4. *perfectus* group (Ribeiro-Costa 1995): *A. anosignatus* (Chevrolat, 1877), *A. bidentatus* Ribeiro-Costa, 1999b, *A. denticulatus* Ribeiro-Costa, 1999b, *A. perfectus* (Sharp, 1885), *A. imperfectus* Kingsolver, 1980. Romero-Nápoles et al. (1996) subsequently proposed the group with the same name composed only by the shared species *A. perfectus*. The species *A. anosignatus* was included in this group probably by an artifact of the methodology used by Ribeiro-Costa (1995). This species differs from the others of the group mainly by the integument color and the absence of teeth surrounding the sclerite complex in the median region of the internal sac of male genitalia (Ribeiro-Costa 1995:33), characters that justify its exclusion from the group. The *perfectus* group is now composed of four species: *A. bidentatus*, *A. denticulatus*, *A. perfectus*, and *A. imperfectus*.
5. *pterocarpae* group (Ribeiro-Costa 1995): *A. pterocarpae* Kingsolver, 1980 and *A. luciae* Ribeiro-Costa, 1999b. Romero-Nápoles et al. (1996) proposed the group with the same name containing only the shared *A. pterocarpae*.

Although there are other groups which we identify as not having disagreements among their species composition, we consider it premature at this moment to merge them.

1. *robiniae* group (Kingsolver 1975) and *robiniae* group (Romero-Nápoles et al. 1996);
2. *profaupar* group (Ribeiro-Costa 1995) and *scutellaris* group (Romero-Nápoles et al. 1996);
3. *megalobus* and *canescens* groups (Ribeiro-Costa 1995) and *championi* group (Romero-Nápoles et al. 1996).

Furthermore, there are different conclusions on group membership based on species that do overlap.

1. *A. acapulcensis* Kingsolver, 1975 (*robiniae* groups);
2. *A. baracoensis* Kingsolver, 1970 (*profaupar* and *scutellaris* groups);
3. *A. cerdanicola* Kingsolver, 1970 (*profaupar* and *scutellaris* groups);
4. *A. championi* (Pic, 1913) (*championi* and *megalobus* groups);
5. *A. robiniae* (*robiniae* groups);
6. *A. scutellaris* (Sharp, 1885) (*profaupar* and *scutellaris* groups) ;
7. *A. teutoniensis* (Ribeiro-Costa & Kingsolver, 1993b) (*canescens* and *championi* groups).

We therefore suggest a future taxonomic revision or a phylogenetic, morphological and/or molecular studies of the groups to which they belong before reconciling membership between the different groups.

## ﻿Discussion

We have been focusing on the Brazilian *Amblycerus* fauna for a long time and with the four more species added here we reach 36 species described by the first author and her collaborators; the total number of Brazilian species encompasses 66 known species and including the new ones of the *sclerolobii* group we record a total of 70 Brazilian species; certainly many more are to be discovered for this country. All host plants known for this species group belong to the Caesalpinoideae clade of the Fabaceae (Bruneau et al. 2024). The tribes Dimorphandreae, Campsiandreae, and Sclerolobieae are closely related lineages that include nine genera with 140–165 species of trees in Neotropical forests (Bruneau et al. 2024); the *Stryphnodendron* clade of the tribe Mimoseae is somewhat more distantly related and also includes forest trees in the Neotropics and is composed of seven genera and 56 species (Bruneau et al. 2024). Trees from neotropical wet forests remain undersampled for their seed beetle diversity, especially when compared to the more easily accessible flora of dry deciduous forests and thorn scrub. The results of this study indicate that the species diversity within these four clades could provide significant opportunities to more thoroughly document the diversity of the genus *Amblycerus* and perhaps particularly the *sclerolobii* species group.

This revision of the *Amblycerus* species groups provides a clear view of the current state of knowledge at this intrageneric taxonomic level. At the beginning of this review we listed 37 groups, and in the end, five groups were merged although two still remain with the same name (*robiniae* group), reduced now to 32 species groups. These groups encompass a total of 96 species of *Amblycerus*, with 26 species remaining to be studied if we consider a total of 122 currently valid species assigned into this genus.

The characterization of *Amblycerus* groups and the definitions of their limits have not been a simple task. The entire data set, which reflects the extensive morphological convergence resulting from the constraints of developing within seeds (Smith-Pardo et al. 2024), makes the identification process a challenge at both the group and species levels. Diagnostic characters are not always the same among groups and variations of characters may occur within the same group. In the *sclerolobii* group, the apically tridentate scutellum is one of the diagnostic characters of the group; however, in *A. truncatus* Ribeiro-Costa, sp. nov., the scutellum is truncate, but the characters of the male genitalia of this species place it in this group. Also, the tridentate scutellum appears in other species groups suggesting that they have evolved independently in different lineages, similar to the patterns of colors on the dorsum. The patterns of integument/pubescence on the dorsum of a group is not always congruent with the male genitalia pattern of sclerites in the internal sac, as we found in *A. manauara* Ribeiro-Costa, 2000 and *A. tachigaliae* Kingsolver, 1976, but it is possible that the male genitalia pattern of sclerites defines a group as mentioned in *A. truncatus* Ribeiro-Costa, sp. nov., even if we found some variability in the pattern of integument/pubescence.

Herein we have contributed to the formal description of groups and its species with well-illustrated male genitalia and color illustrations of external characters to avoid misidentification. Both contributions of Ribeiro-Costa (1995) and Romero-Nápoles et al (1996) did not include colored images and the second one did not even provide illustrations of the pattern on dorsum. In this paper, we include not only colored images and detailed drawings of male genitalia but comparative characters useful for phylogenetic morphological study or even discussion of results based on molecular data analysis as we agree that all taxonomically proposed groups should be tested through repeatable phylogenetic analyses.

## Supplementary Material

XML Treatment for Amblycerus truncatus

XML Treatment for Amblycerus falcorostrus

XML Treatment for Amblycerus biacutus

XML Treatment for Amblycerus morsei

XML Treatment for Amblycerus sclerolobii

XML Treatment for Amblycerus kingsolveri

XML Treatment for Amblycerus marinonii

XML Treatment for Amblycerus manauara

XML Treatment for Amblycerus tachigaliae

XML Treatment for Amblycerus bicolor

## ﻿Additional information

### Author ORCIDs

Cibele Stramare Ribeiro-Costa https://orcid.org/0000-0001-6527-6460

Geoffrey E. Morse https://orcid.org/0000-0002-5763-8246

### Data availability

All of the data that support the findings of this study are available in the main text.
